# Antibacterial activity of *Bacillus* species-derived surfactin on *Brachyspira hyodysenteriae* and *Clostridium perfringens*

**DOI:** 10.1186/s13568-019-0914-2

**Published:** 2019-11-21

**Authors:** Yi-Bing Horng, Yu-Hsiang Yu, Andrzej Dybus, Felix Shih-Hsiang Hsiao, Yeong-Hsiang Cheng

**Affiliations:** 1 Department of Biotechnology and Animal Science, National Ilan University, Yilan City, 26047 Yilan County Taiwan; 2 Laboratory of Molecular Cytogenetics, Department of Genetics, Faculty of Biotechnology and Animal Husbandry, West Pomeranian University of Technology, Szczecin, Poland; 3 Department of Animal Science and Biotechnology, Tunghai University, Taichung, Taiwan

**Keywords:** Antibacterial activity, *Bacillus licheniformis*, *Brachyspira hyodysenteriae*, *Clostridium perfringens*, Surfactin

## Abstract

Swine dysentery and necrotic enteritis are a bane to animal husbandry worldwide. Some countries have already banned the use of antibiotics as growth promoters in animal production. Surfactin is a potential alternative to antibiotics and antibacterial agents. However, the antibacterial activity of *Bacillus* species-derived surfactin on *Brachyspira hyodysenteriae* and *Clostridium perfringens* are still poorly understood. In the current study, the antibacterial effects of surfactin produced from *Bacillus subtilis* and *Bacillus licheniformis* on *B. hyodysenteriae* and *C. perfringens* were evaluated. Results showed that multiple surfactin isoforms were detected in *B. subtilis*, while only one surfactin isoform was detected in *B. licheniformis* fermented products. The surfactin produced from *B. subtilis* exhibited significant antibacterial activity against *B. hyodysenteriae* compared with surfactin produced from *B. licheniformis*. *B. subtilis*-derived surfactin could inhibit bacterial growth and disrupt the morphology of *B. hyodysenteriae*. Furthermore, the surfactin produced from *B. subtilis* have the highest activity against *C. perfringens* growth. In contrast, *B. licheniformis* fermented product-derived surfactin had a strong bacterial killing activity against *C. perfringens* compared with surfactin produced from *B. subtilis*. These results together suggest that *Bacillus* species-derived surfactin have potential for development as feed additives and use as a possible substitute for antibiotics to prevent *B. hyodysenteriae* and *C. perfringens*-associated disease in the animal industry.

## Introduction

Swine dysentery (SD) caused by *Brachyspira hyodysenteriae* is a highly contagious disease of grower and finisher pigs. SD causes severe mucohemorrhagic diarrhea, resulting in decreased feed efficiency and increased morbidity (Alvarez-Ordóñez et al. 2013). Antibiotics, such as tiamulin and carbadox, are widely used for prevention and treatment of SD due to their relatively short withdrawal periods and the high susceptibility of *Brachyspira* species (van Duijkeren et al. 2014). However, accumulating evidence suggests that *B. hyodysenteriae* resistance to antibiotics is commonly observed (Mirajkar et al. 2016; Hampson et al. 2019).

Necrotic enteritis (NE) caused by *Clostridium perfringens* is characterized by high mortality in poultry with bloody diarrhea, and sudden death (Kaldhusdal and Løvland 2000; Lee et al. 2011). Predisposing factors for necrotic enteritis have been proposed, such as coccidiosis, stress, and nutritional factors (M’Sadeq et al. 2015). Bacitracin has been commonly used worldwide as an antibiotic growth promoter for prophylactic treatment of *C. perfringens*-induced NE in poultry (M’Sadeq et al. 2015; Caly et al. 2015). However, the European Union has already banned antibiotics used in animal production, resulting in increased NE outbreaks in broilers in European countries (Van der Sluis 2000; Van Immerseel et al. 2004).

Several antibiotic alternatives have been proposed for prophylactic treatment of diseases, such as probiotics (Abudabos et al. 2013, 2017; Cheng et al. 2018, 2019). In recent years, fermented products produced from *Bacillus subtilis* and *Bacillus licheniformis* have gained attention as probiotic supplements in animal feed due to the production of thermostable and low pH resistant spores (Cheng et al. 2018; Lin et al. 2019). *B. subtilis* and *B. licheniformis* have been identified from the gastrointestinal tract of broilers with antimicrobial activity (Barbosa et al. 2005; Cheng et al. 2018; Lin et al. 2019). Furthermore, it has been demonstrated that *Bacillus* species are able to produce a variety of antimicrobial peptides (Sumi et al. 2015). The surfactin, *Bacillus*-derived cyclic lipopeptide, is an important antimicrobial peptide with antibacterial activity through disruption of the bacterial membrane (Carrillo et al. 2003; Chen et al. 2008). However, whether *B. subtilis* and *B. licheniformis*-derived surfactin has antibacterial activity against *B. hyodysenteriae* and *C. perfringens* still remain to be elucidated.

SD and NE are prevalent and important enteric diseases, which lead to enormous economic losses in animal husbandry worldwide (Van der Sluis 2000; Timbermont et al. 2011; Card et al. 2018). The restrictions placed on the prophylactic use of antibiotics in animal production and the prevalence of multidrug resistant bacteria, mean that alternative solutions to prevent and treat SD and NE are an urgent unmet need in animal husbandry. In the present study, we investigated the antibacterial activity of *Bacillus* species-derived surfactin on *B. hyodysenteriae* and *C. perfringens*. The results provide valuable knowledge for understanding the antibacterial potential of surfactin-producing *Bacillus* species as a substitute for antibiotics.

## Materials and methods

All experiments were performed in accordance with the approved guidelines.

### Preparation of *Bacillus* species-derived surfactin

*Bacillus subtilis*-derived surfactin is a commercially available product (Sigma-Aldrich, St. Louis, MO, USA). The *B. licheniformis*-derived surfactin was extracted from solid-state fermented products. The *B. licheniformis* was purchased from the Food Industry Research and Development Institute (ATCC 12713, Hsinchu, Taiwan). Details of *B. licheniformis*-fermented product preparation were as described in a previous study (Lin et al. 2019). After thawing, the *B. licheniformis* was inoculated into an Erlenmeyer flask containing tryptic soy broth (TSB; Sigma-Aldrich, St. Louis, MO, USA) and incubated at 30 °C for 18 h with shaking at 160 rpm. The solid-state fermentation substrates in a space bag were inoculated with 4% (v/w) inoculum of *B. licheniformis* and incubated at 30 °C in a chamber with free oxygen and relative humidity above 80% for 6 days. The fermented products were dried at 50 °C for 2 days and homogenized by mechanical agitation. The fermented powder was then stored at 4 °C prior to analysis.

### Extraction and analysis of surfactin

The supernatant of *B. licheniformis*-fermented products was adjusted to pH 2.0 with concentrated HCl and incubated overnight at 4 °C. The precipitate was dissolved in distilled water and extracted with methanol. The mixture was shaken vigorously and the organic phase was concentrated at reduced pressure at 40 °C. The extract was further filtered using a syringe filter with a 0.22 μm membrane. The surfactin concentration in the filtrate was quantified and measured using high performance liquid chromatography. The SPD-10A system (Shimadzu, Columbia, MD, USA) with a programmable UV detector (10A VP, Shimadzu, Columbia, MD, USA) and a reverse phase RP-18 column (LiChrospher 100 RP-18 endcapped, 5 μm) was used throughout the experiments. Samples were injected into the HPLC column. The mobile phase consisted of 3.8 mM trifluoroacetic acid: acetonitrile (20:80, v/v). The flow rate was 1 ml/min. Surfactin was determined at a wavelength of 210 nm by use of a UV detector. The recorder was set to 30 min.

### Test bacteria

*Brachyspira hyodysenteriae* (ATCC 27164) was cultured in brain heart infusion broth (BHIB, EMD Millipore, Danvers, MA, USA). *Clostridium perfringens* (ATCC13124) was cultured in Gifu anaerobic medium (GAM; Sigma-Aldrich, St. Louis, MO, USA). After two successive transfers of the test organisms in specific culture media, the activated culture was inoculated into specific culture media for further quantification.

### Confocal microscopy

BHIB containing different dilutions of *B. subtilis*-derived surfactin (concentration range from 9.31 to 500 μg/ml) was incubated with *B. hyodysenteriae* (10^4^ CFU/ml) at 42 °C for 0.5 h and 1 h, respectively. GAM containing different dilutions of *B. subtilis*-derived surfactin (concentration range from 7.8 to 500 μg/ml) was incubated with *C. perfringens* (10^4^ CFU/ml) at 37 °C for 0.5 h and 1 h, respectively. After incubation, SYBR Green I, propidium iodide and Hoechst 33342 were added to each well and then stained at 37 °C for 30 min in the dark. The stained samples were examined under a confocal laser-scanning microscope (ZEISS LSM800, Jena, Germany). For the detection of the SYBR Green I stained cells, 497 nm excitation wavelengths and 520 nm emission wavelengths were used. For the detection of the propidium iodide stained cells, 530 nm excitation wavelengths and 620 nm emission wavelengths were applied. For the detection of the Hoechst 33342 stained cells, 335 nm excitation and 460 nm emission were used. The number of live (green) or dead (red) cells were viewed and counted manually using the ImageJ software.

### Agar-well diffusion assay

The *B. subtilis* and *B. licheniformis*-derived surfactin were serially diluted (concentration range from 7.8 to 500 μg/ml) and transferred into a well in tryptic soy agar (TSB; Sigma-Aldrich, St. Louis, MO, USA) containing 5% goat blood (collected from healthy goat in experimental farm of National Ilan University) and *B. hyodysenteriae* (1 × 10^4^ CFU/ml), or Gifu anaerobic medium agar (GAM agar; Sigma-Aldrich, St. Louis, MO, USA) containing the *C. perfringens* (1 × 10^4^ CFU/ml), respectively. For measurement of the inhibition zone of *B. hyodysenteriae*, the plates were incubated at 42 °C for 24 h. For measurement of the inhibition zone of *C. perfringens*, the plates were incubated at 37 °C for 24 h. The antibacterial activity of surfactin was determined by measuring the diameter of this zone of inhibition using the ImageJ software (NIH, Bethesda, MD, USA).

### Antibacterial assay

The antibacterial activity of surfactin was studied by employing a microdilution method. The *B. subtilis* and *B. licheniformis*-derived surfactin were serially diluted (concentration range from 31.25 to 500 μg/ml). One hundred microliters of BHIB containing different dilutions were distributed in 96-well plates. Each well was inoculated with *B. hyodysenteriae* (10^4^ CFU/ml) and incubated anaerobically at 42 °C for 0.5 h and 1 h, respectively. One hundred microliters of GAM containing different dilutions were distributed in 96-well plates. Each well was inoculated with *C. perfringens* (10^4^ CFU/ml) and incubated anaerobically at 37 °C for 0.5 h and 1 h, respectively. All experiments were performed in triplicate. The bacterial survival analyzed by turbidity was detected using optical density at 600 nm (EMax Plus Microplate Reader, Sunnyvale, CA, USA). Data were normalized by absorbance and by negative controls, with untreated sample set at 100% survival.

### Scanning electron microscopy

BHIB containing different dilutions of *B. subtilis*-derived surfactin (concentration range from 10 to 250 μg/ml) was incubated with *B. hyodysenteriae* (10^4^ CFU/ml) at 42 °C for 1 h, respectively. The cells were fixed with 2.5% glutaraldehyde in 50 mM potassium phosphate buffer (pH 6.8) overnight at 4 °C, and then washed three times with 50 mM potassium phosphate buffer (pH 6.8). Dehydration was performed in different concentrations of ethanol (50–100%) for 10 min each and the samples were dried in a critical point drier (CPD 030, Bal-Tec AG, Balzers, Liechtenstein). Specimens were coated under vacuum with gold: palladium (60:40) in a sputter coater (Bal-Tec AG, Balzers, Liechtenstein), and examined using a scanning electron microscope (JSM-6300, JEOL Ltd., Tokyo, JAPAN).

### Statistical analysis

Data were analyzed by one‐way ANOVA using the GLM procedure of the SAS software package (Version 9.4; SAS Institute, Cary, NC). Replicates were considered the experimental units. Results were expressed as mean ± SD. Means were compared by employing Tukey’s HSD test at a significance level of *P* < 0.05.

## Results

### Identification of surfactin from *B. subtilis* and *B. licheniformis*

**Fig. 1:**
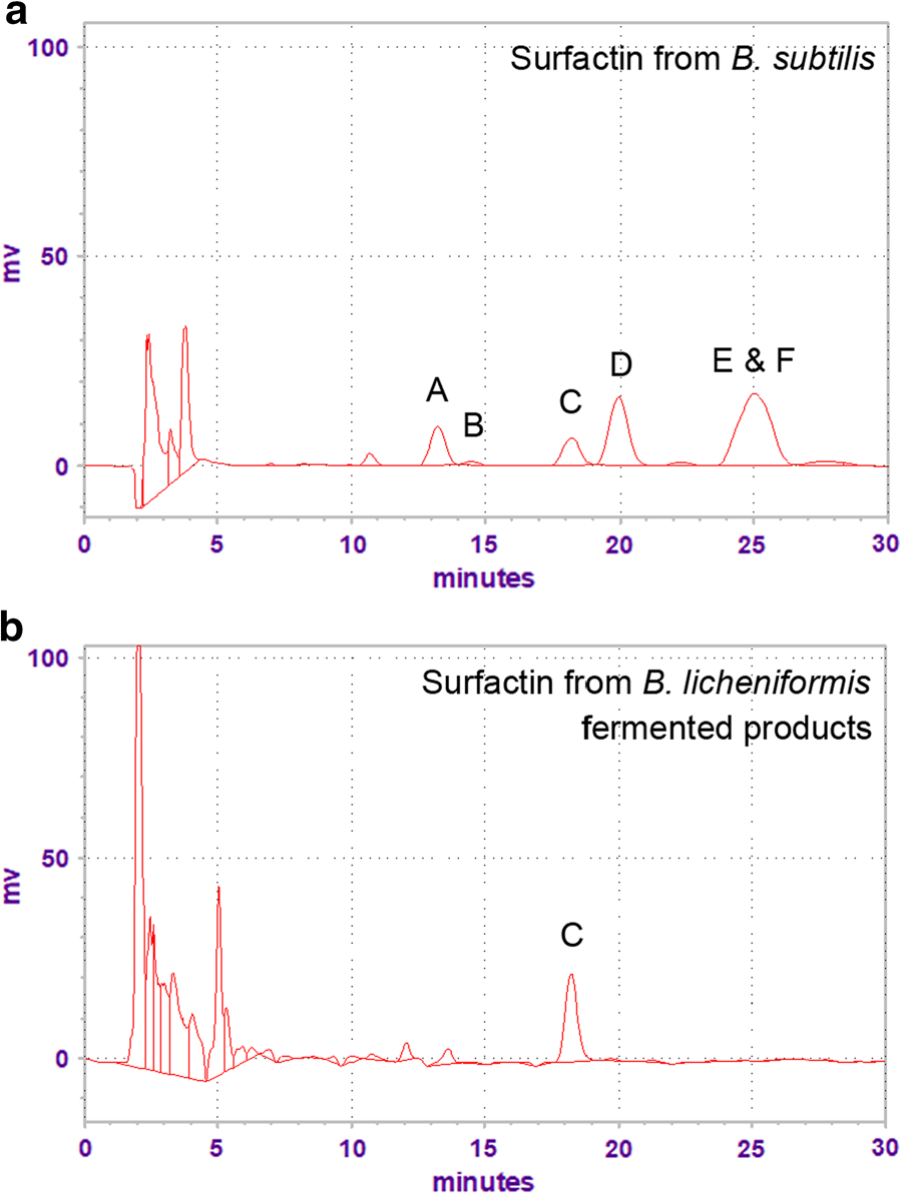
HPLC chromatogram of surfactin. **a** The chromatographic peak of *B. subtilis*-derived surfactin and **b** *B. licheniformis*-fermented product-derived surfactin

The chromatographic peak of *B. subtilis* and *B. licheniformis*-derived surfactin is presented in Fig. 1. Results showed that multiple retention peaks were detected in the *B. subtilis*-derived surfactin (Fig. 1a). However, only a single peak of surfactin from *B. licheniformis* fermented products was detected which had a chromatogram identical to surfactin C in *B. subtilis*-derived surfactin (Fig. 1b).

### Antibacterial activity of *Bacillus* species-derived surfactin on *B. hyodysenteriae*

**Fig. 2:**
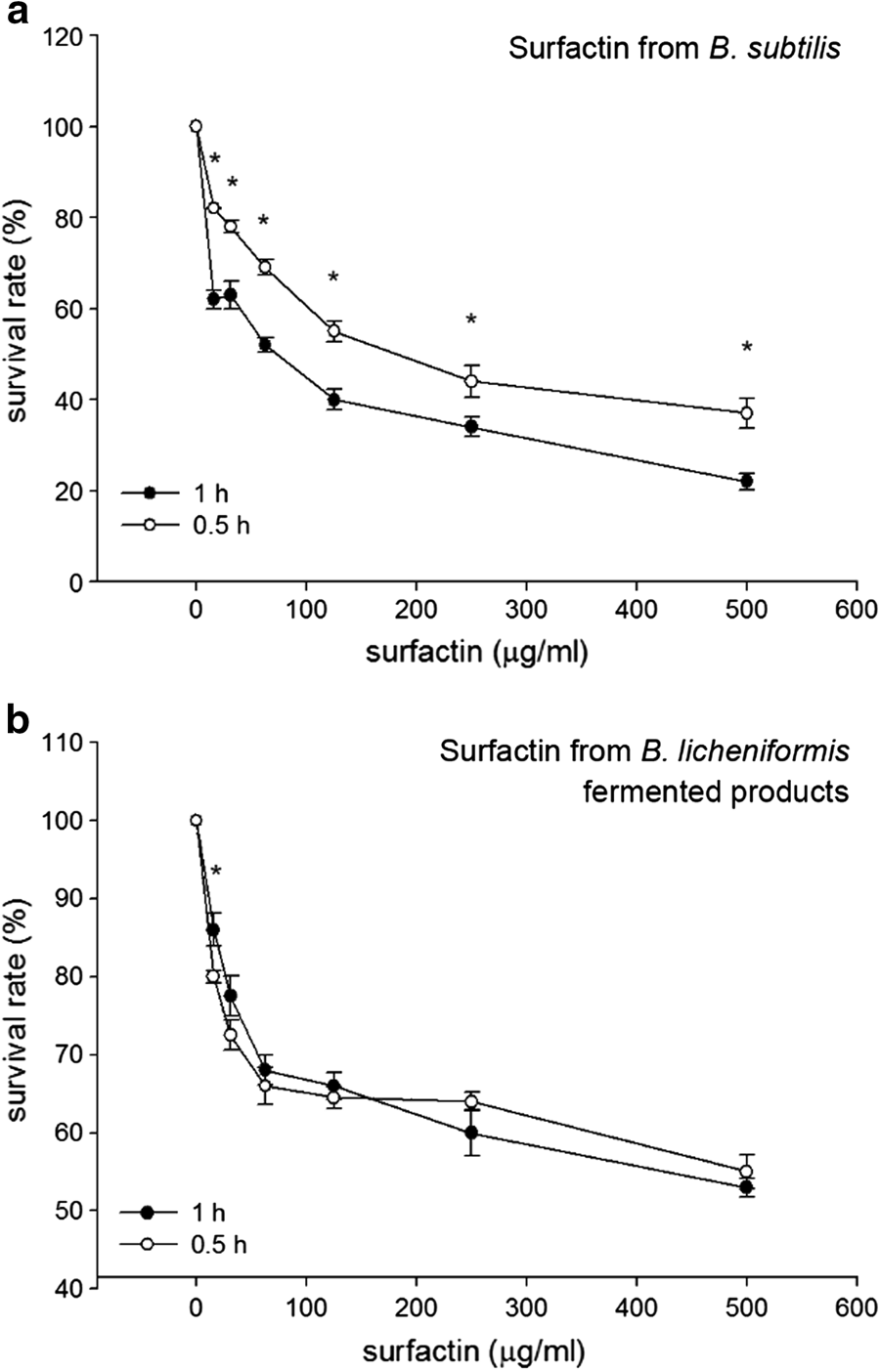
Killing activity of surfactin against *B. hyodysenteriae*. **a** *B. hyodysenteriae* were incubated with different concentrations (9.31, 18.63, 37.25, 62.5, 125, 250, 500 μg/ml) of the *B. subtilis*-derived surfactin for 0.5 and 1 h. **b** *B. hyodysenteriae* were incubated with different concentrations (9.31, 18.63, 37.25, 62.5, 125, 250, 500 μg/ml) of the *B. licheniformis*-fermented product-derived surfactin for 0.5 and 1 h. The error bars represent the mean ± standard deviations from triplicate assays (n = 3). *P < 0.05 comparing data of 0.5 h versus 1 h

**Fig. 3:**
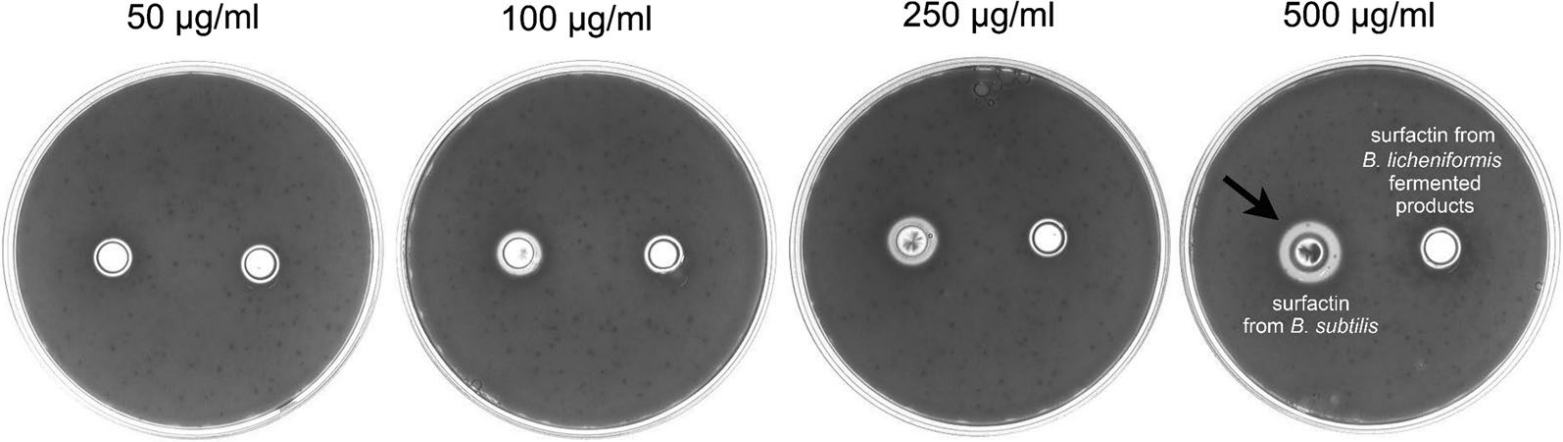
Growth inhibitory zone of surfactin against *B. hyodysenteriae* using agar-well diffusion assay. *B. hyodysenteriae* were grown in the different concentrations (50, 100, 250, 500 μg/ml) of surfactin produced from *B. subtilis* (left) and *B. licheniformis*-fermented products (right) for 24 h. The black arrow indicates inhibition zone. Three experiments were conducted, and one representative result is presented

**Fig. 4:**
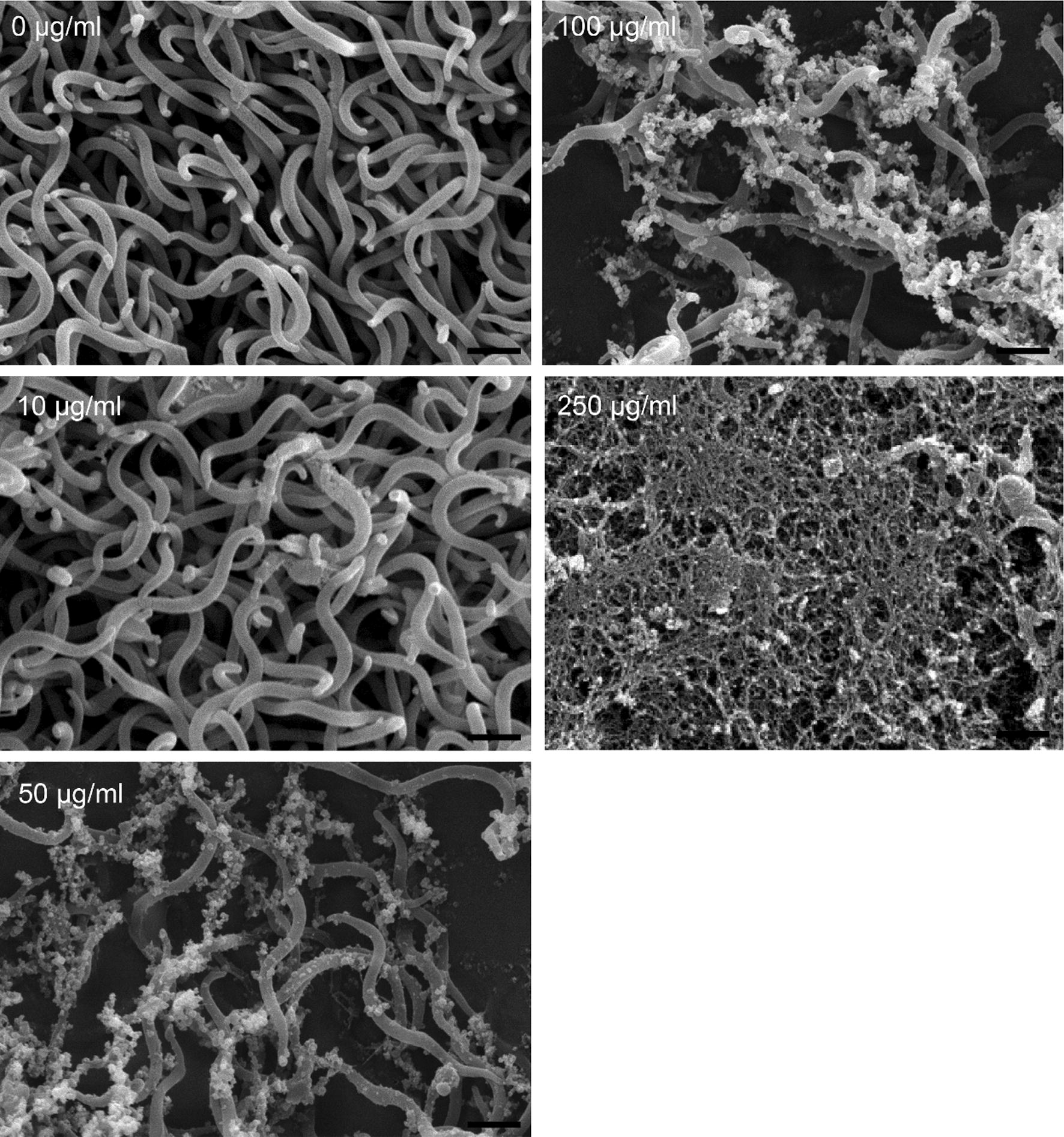
Scanning electron microscope images of *B. hyodysenteriae* illustrating the antibacterial activity of different concentrations (10, 50, 100, 250 μg/ml) of the commercial surfactin after incubating for 1 h. Three experiments were conducted, and one representative result is presented

Results of confocal microscopy examinations showed that *B. subtilis*-derived surfactin is able to cause the death of *B. hyodysenteriae* in a dose dependent manner (Fig. 2a). A longer duration (1 h) of *B. subtilis*-derived surfactin treatment could increase the death rate of *B. hyodysenteriae* (*P* < 0.05, Fig. 2a). Similarly, the surfactin from *B. licheniformis* fermented products also caused the death of *B. hyodysenteriae* in a dose dependent manner (Fig. 2b). Duration of treatment of *B. licheniformis*-derived surfactin did not affect the death rate of *B. hyodysenteriae* except with 9.31 μg/ml treatment (Fig. 2b). Relative to the surfactin from *B. licheniformis* fermented products, *B. subtilis*-derived surfactin had the highest antibacterial activity against *B. hyodysenteria* (Fig. 2a, b). Results of bacterial survival analysis by turbidity examinations showed that *B. subtilis*-derived surfactin significantly caused the death of *B. hyodysenteriae* in a dose dependent manner after 0.5 h treatment (*P* < 0.05, Table 1). However, prolonged treatment (1 h) of different concentrations of *B. subtilis*-derived surfactin did not further promote the death of *B. hyodysenteriae*. Results of agar-well diffusion assay revealed that *B. subtilis*-derived surfactin exhibited a significant inhibition zone compared with *B. licheniformis* fermented product-derived surfactin (Fig. 3). The results of quantification of the inhibition zone are presented in Table 2. Results showed that the *B. subtilis*-derived surfactin caused the inhibition of *B. hyodysenteriae* growth in a dose dependent manner (*P* < 0.05). Similarly, *B. licheniformis* fermented products also had a dose dependent inhibitory effect on *B. hyodysenteriae* growth (*P* < 0.05). The zone of inhibition of *B. subtilis*-derived surfactin on *B. hyodysenteriae* growth was greater than *B. licheniformis* fermented products-derived surfactin (*P* < 0.05). Furthermore, results of scanning electron microscopy showed that *B. subtilis*-derived surfactin could disrupt the morphology of *B. hyodysenteriae* in a dose dependent manner after 1 h treatment compared with the untreated group (Fig. 4).

**Table 1:** Percentage survival rate of *B. hyodysenteriae* in response to *B. subtilis*-derived surfactin treatment

Concentration (μg/ml)	0.5 h		1 h	
	Survival rate (%)			
	Mean	SD	Mean	SD
0	100^a,d^	0	100^a^	0
10	95.2^a^	5.1	92.4^a^	3.8
50	63.7^b^	8.3	53.1^b^	4.6
100	42.5^bc^	3.5	38.2^b^	7.4
250	12.8^c^	6.7	12.4^c^	5.2

^a–c^Means within a column with no common superscript are significantly different (*P *<0.05)

^d^Values are expressed as mean ± SD (n = 3)

**Table 2:** Measurement of zone of inhibition of surfactin on *B. hyodysenteriae* growth at different concentrations

Concentration (μg/ml)	Surfactin from *B. subtilis*		Surfactin from *B. licheniformis* fermented products	
	Zone of inhibition (cm)			
	Mean	SD	Mean	SD
50	0.3^a,x,d^	0.02	0.2^a,y^	0.01
100	0.6^b,x^	0.03	0.2^a,y^	0.01
250	0.9^bc,x^	0.02	0.4^b,y^	0.01
500	1.4^c,x^	0.04	0.5^b,y^	0.02

^a–c^Means within a column with no common superscript are significantly different (*P *<0.05)

^x–y^Means within a row with no common superscript are significantly different (*P *<0.05)

^d^Values are expressed as mean ± SD (n = 3)

### Antibacterial activity of *Bacillus* species-derived surfactin on *C. perfringens*

**Fig. 5:**
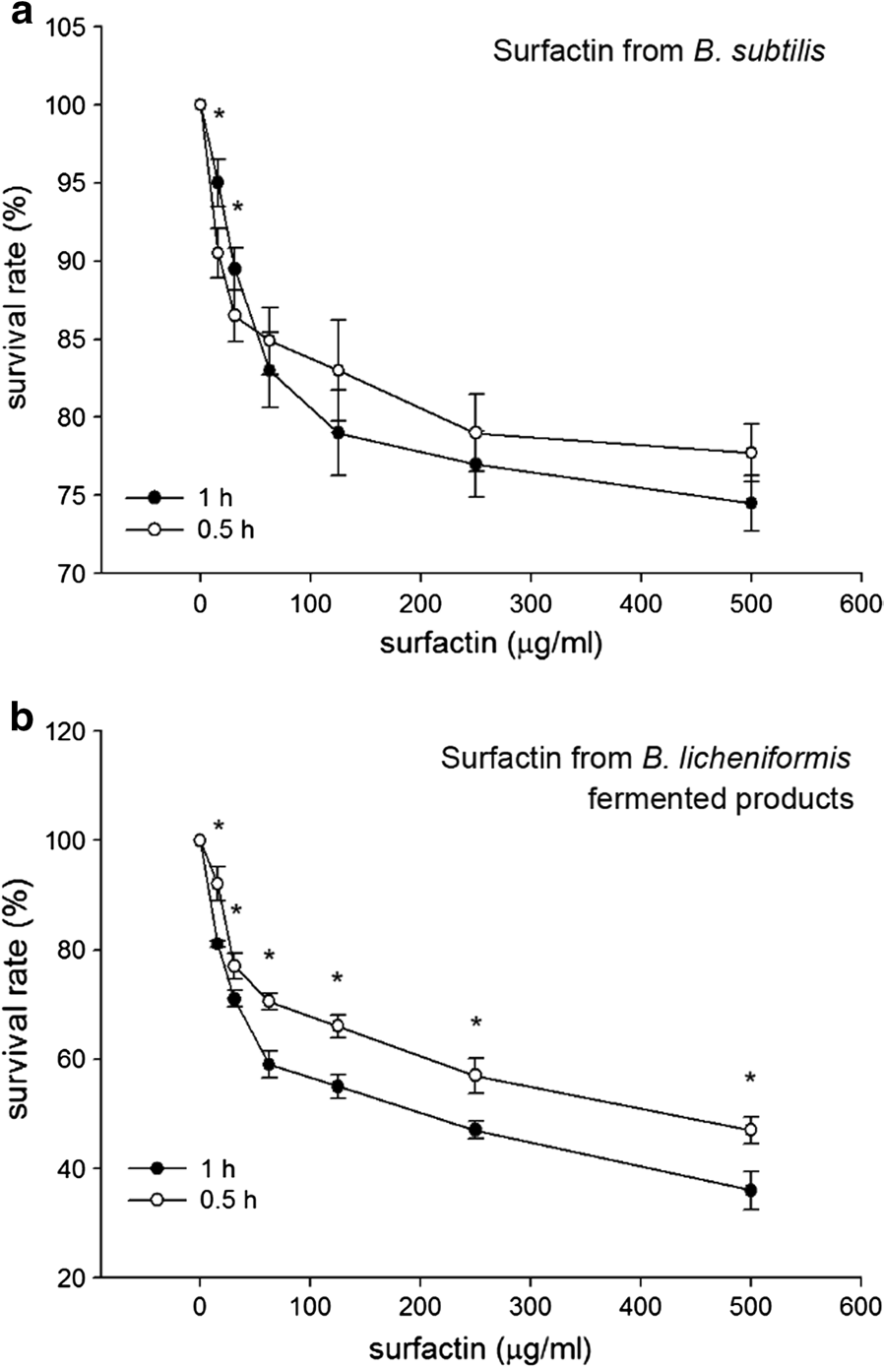
Killing activity of surfactin against *C. perfringens*. **a** *C. perfringens* were incubated with different concentrations (7.8, 15.62, 31.25, 62.5, 125, 250, 500 μg/ml) of the *B. subtilis*-derived surfactin for 0.5 and 1 h. **b** *C. perfringens* were incubated with different concentrations (7.8, 15.62, 31.25, 62.5, 125, 250, 500 μg/ml) of the *B. licheniformis*-fermented products-derived surfactin for 0.5 and 1 h. The error bars represent the mean ± standard deviations from triplicate assays (n = 3). *P < 0.05 comparing data of 0.5 h versus 1 h

**Fig. 6:**
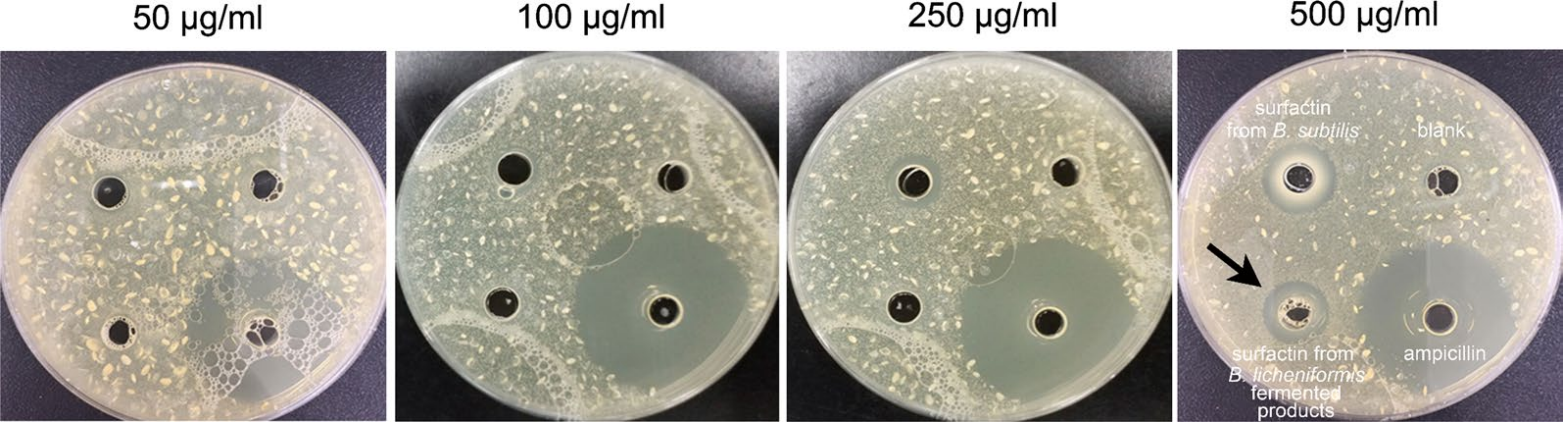
Growth inhibitory zone of surfactin against *C. perfringens* using agar-well diffusion assay. *C. perfringens* were grown in the different concentrations (50, 100, 250, 500 μg/ml) of the surfactin produced from *B. subtilis* (top left) and *B. licheniformis*-fermented products (bottom left), blank (top right), and ampicillin (bottom right) for 24 h. The black arrow indicates the inhibition zone. Three experiments were conducted, and one representative result is presented

Results of confocal microscopy examinations showed that *B. subtilis*-derived surfactin is able to cause the death of *C. perfringens* in a dose dependent manner (Fig. 5a). A longer duration (1 h) of *B. subtilis*-derived surfactin treatment could increase the death rate of *C. perfringens* except at 7.8 and 15.62 μg/ml (*P* < 0.05, Fig. 5a). The surfactin from *B. licheniformis* fermented products also caused the death rate of *C. perfringens* to increase in a dose dependent manner (Fig. 5b). Similarly, a longer (1 h) duration of *B. licheniformis* fermented product-derived surfactin treatment could increase the death of *C. perfringens* (*P* < 0.05, Fig. 5b). Relative to *B. subtilis*-derived surfactin, surfactin from *B. licheniformis* fermented products had the highest antibacterial activity against *C. perfringens* (Fig. 5a, b). Results of bacterial survival analysis by turbidity examinations showed that *B. subtilis*-derived surfactin significantly caused the death of *C. perfringens* in a dose dependent manner after 0.5 h treatment (*P* < 0.05, Table 3). Prolonged treatment (1 h) of different concentrations of *B. subtilis*-derived surfactin could further promote the death of *C. perfringens* (*P* < 0.05). Results of agar-well diffusion assay showed that *B. subtilis*-derived surfactin exhibited a significant inhibition zone (250 μg/ml and 500 μg/ml) compared with *B. licheniformis* fermented product-derived surfactin (Fig. 6). The results of quantification of the inhibition zone are presented in Table 4. Results showed that *B. subtilis*-derived surfactin caused the inhibition of *C. perfringens* growth in a dose dependent manner (*P* < 0.05). Similarly, *B. licheniformis* fermented product-derived surfactin also had a dose dependent inhibitory effect on *C. perfringens* growth (*P *< 0.05). In addition, the zone of inhibition of *B. subtilis*-derived surfactin on *C. perfringens* growth was greater than *B. licheniformis* fermented product-derived surfactin (*P* < 0.05).

**Table 3:** Percentage survival rate of the *C. perfringens* in response to *B. subtilis*-derived surfactin treatment

Concentration (μg/ml)	0.5 h		1 h	
	Survival rate (%)			
	Mean	SD	Mean	SD
0	98.0^a,x,d^	2.8	95^a,x^	2.2
31.25	89.1^a,x^	9.2	83^ab,x^	3.8
62.5	81.3^ab,x^	10.3	72^b,x^	3.4
125	72.1^ab,x^	1.2	57^c,y^	2.1
250	68.4^b,x^	5.4	52^c,y^	7.9

^a–c^Means within a column with no common superscript are significantly different (*P *<0.05)

^x–y^Means within a row with no common superscript are significantly different (*P *<0.05)

^d^Values are expressed as mean ± SD (n = 3)

**Table 4:** Measurement of zone of inhibition of surfactin on *C. perfringens* growth at different concentrations

Concentration (μg/ml)	Surfactin from *B. subtilis*		Surfactin from *Bacillus licheniformis* fermented products	
	Zone of inhibition (cm)			
	Mean	SD	Mean	SD
31.25	0.6^a,x,e^	0.04	0.5^a,y^	0.00
62.5	0.6^a,x^	0.02	0.5^a,y^	0.01
125	0.9^b,x^	0.02	0.6^b,y^	0.03
250	1.0^c,x^	0.05	0.6^b,y^	0.03
500	1.3^d,x^	0.03	1.1^c,y^	0.03

^a–d^Means within a column with no common superscript are significantly different (*P *<0.05)

^x–y^Means within a row with no common superscript are significantly different (*P *<0.05)

^e^Values are expressed as mean ± SD (n = 3)

## Discussion

In this study, we demonstrated that surfactin either from *B. subtilis* or *B. licheniformis* fermented products is able to inhibit the growth of *B. hyodysenteriae* and *C. perfringens*. The *B. subtilis*-derived surfactin exhibited the greatest bacterial killing activity against *B. hyodysenteriae*. In contrast, *B. licheniformis* fermented product-derived surfactin had the highest bacterial killing activity against *C. perfringens*.

Surfactin as a secondary metabolite was first found in *B. subtilis* and consists of multiple isoforms (Haddad et al. 2008; Sousa et al. 2014; Sumi et al. 2015). The structure of surfactin is a seven amino acid peptide loop and a hydrophobic fatty acid chain (Kowall et al. 1998). It has been reported that surfactin has a broad spectrum of antimicrobial activity against pathogenic microbes (Chen et al. 2008; Sumi et al. 2015). Our previous study demonstrated that *B. subtilis*-fermented products with the highest surfactin concentration show antibacterial activity against *Escherichia coli*, *Staphylococcus aureus*, *Salmonella typhimurium* and *C. perfringens* in vitro (Cheng et al. 2018). A previous study showed that surfactin produced by different strains of *B. subtilis* exhibited diverse antibacterial activity (Sabaté and Audisio 2013). Since most *Bacillus* species can produce two or three types of lipopeptides (Sumi et al. 2015). It has been demonstrated that commercial surfactin (Sigma-Aldrich), produced from *B. subtilis*, has six isoforms (Sousa et al. 2014). Similarly, we also found that multiple isoforms of surfactin were identified in the commercial surfactin in the present study. Environmental and nutritional conditions are critical factors for determining the surfactin isoforms of *B. subtilis* (Peypoux and Michel 1992; Kowall et al. 1998). Thus, it is possible that a proportion of surfactin isoforms might exhibit differential antibacterial property in *B. subtilis*. However, the effect of surfactin isoforms isolated from *Bacillus* species and any interaction between surfactin isoforms on antibacterial activity have not been studied.

In addition to *B. subtilis*, *B. licheniformis* also has the ability to synthesize antimicrobial substances, such as surfactin (Pecci et al. 2010; Sumi et al. 2015). This antimicrobial substance shows antibacterial activity against a wide range of Gram-positive bacteria, such as *Listeria monocytogenes* and Methicillin-resistant *S. aureus*, but does not cause hemolysis or inhibit the growth of Gram-negative bacteria (Dischinger et al. 2009; Abdel-Mohsein et al. 2011). To date, the isoforms of surfactin and antibacterial activity in *B. licheniformis* have rarely been studied. Here, we demonstrated for the first time that the major isoform of surfactin in *B. licheniformis* was surfactin C. Furthermore, surfactin C extracted from *B. licheniformis* fermented products had antibacterial activity against *B. hyodysenteriae* and *C. perfringens*. Our previous study has demonstrated that *B. licheniformis*-fermented products were able to inhibit the growth of *C. perfringens* and *S. aureus* in vitro (Lin et al. 2019). Taken together, these findings indicate that the antibacterial activity of *B. licheniformis* might be mediated by producing surfactin C.

*Brachyspira*-associated diseases, such as SD, are effectively treated with macrolides, lincosamides, and carbadox (Hampson et al. 2019). Morbidity in finishing pigs with SD ranges from 50 to 90% (Burrough 2016). Trends of decreasing susceptibility among *B. hyodysenteriae* for the macrolides and pleuromutilins have been well documented (Rugna et al. 2015; Mirajkar et al. 2016). It has been reported that *B. subtilis* is able to inhibit the growth of *B. hyodysenteriae* in vitro (Klose et al. 2010). Here, we further demonstrated that commercial surfactin produced from *B. subtilis* and *B. licheniformis* fermented product-derived surfactin had antibacterial activity against *B. hyodysenteriae*. Furthermore, the morphology of *B. hyodysenteriae* was disrupted after *B. subtilis*-derived surfactin treatment. Several antimicrobial mechanisms of surfactin have been proposed and characterized, including insertion into lipid bilayers, membrane solubilization, destabilization of membrane permeability by channel formation and chelating of mono-and divalent cations (Seydlová and Svobodová 2008). However, the precise mechanisms through which surfactin exerts antibacterial activity on *B. hyodysenteriae* need further investigation.

Our previous study demonstrated that *B. subtilis* and *B. licheniformis*-fermented products can inhibit the growth of *C. perfringens* in vitro (Cheng et al. 2018; Lin et al. 2019). We further found that surfactin isolated from *B. licheniformis*-fermented products exerts antibacterial activity against *C. perfringens*, implying that the most important antibacterial factor is surfactin in *B. licheniformis*-fermented products. Interestingly, differential antibacterial activity against *C. perfringens* between surfactin from *B. subtilis* and *B. licheniformis* was demonstrated in the present study. Commercial surfactin extracted from *B. subtilis* consists of multiple isoforms, while a sole isoform of surfactin (C) was identified in *B. licheniformis*-fermented products; however, whether there is any interaction among the surfactin isoforms which affects antibacterial activity remains to be elucidated.

In conclusion, this paper provides evidence that surfactin has antibacterial activity against *B. hyodysenteriae* and *C. perfringens*. The surfactin produced from *B. subtilis* have the highest activity against *B. hyodysenteriae* and *C. perfringens* growth. The *B. subtilis*-derived surfactin exhibited better bacterial killing activity against *B. hyodysenteriae*. In contrast, *B. licheniformis* fermented product-derived surfactin showed stronger bacterial killing activity against *C. perfringens*.

## Data Availability

Please contact to the authors for all request.
